# Sexual Dimorphism and Aging Effects on Pituitary Gonadotroph Structure and Secretion in Dromedary Camels: Immunohistochemical and Ultrastructural Insights

**DOI:** 10.3390/vetsci13080813

**Published:** 2026-08-17

**Authors:** Shaukat Ali Shaukat Jaspal, Hanan Al-Khalaifah, Muhammad Mubashar Shaukat, Robina Shaukat, Tahmina Shaukat, Shabana Naz, Rifat Ullah Khan, Vincenzo Tufarelli, Caterina Losacco, Luciana Rossi, Mutassim M. Abdelrahman

**Affiliations:** 1 Institute of Zoology, University of the Punjab, Lahore 54000, Pakistanmubashar.jaspal1221@gmail.com (M.M.S.);; 2 Environment and Life Sciences Research Center, Kuwait Institute for Scientific Research, Safat 13001, Kuwait; hkhalifa@kisr.edu.kw; 3 Department of Zoology, Wildlife and Fisheries, University of Agriculture, Faisalabad 38000, Pakistan; teena.jaspal@gmail.com; 4 Department of Zoology, Government College University, Faisalabad 38000, Pakistan; 5 Physiology Lab, College of Veterinary Sciences, Faculty of Animal Husbandry & Veterinary Sciences, The University of Agriculture, Peshawar 25000, Pakistan; 6 Section of Veterinary Science and Animal Production, Department of Precision and Regenerative Medicine and Jonian Area (DiMePRe-J), University of Bari Aldo Moro, s.p. Casamassima km 3, 70010 Valenzano, Italy; vincenzo.tufarelli@uniba.it (V.T.); caterina.losacco@uniba.it (C.L.); 7 Department of Veterinary Medicine and Animal Sciences (DIVAS), University of Milan, 20133 Milan, Italy; luciana.rossi@unimi.it; 8 Department of Animal Production, College of Food and Agriculture Science, King Saud University, Riyadh 11451, Saudi Arabia; amutassim@ksu.edu.sa

**Keywords:** aging, sexual dimorphism, endocrine cells, reproductive physiology, ultrastructure, gonadal function, dromedary camel

## Abstract

This study investigated age- and sex-related variations in pituitary gonadotroph morphometry and serum luteinizing hormone (LH) and follicle-stimulating hormone (FSH) concentrations in 90 clinically healthy dromedary camels (*Camelus dromedarius*). Animals were grouped into three age categories (2–4, 5–10, and ≥11 years), with equal representation of males and females. The precise reproductive status of the female camels (e.g., estrous stage or pregnancy status) was not determined and should be considered when interpreting the endocrine findings. Immunocytochemical analysis revealed significant age- and sex-related differences in gonadotroph number and morphometric characteristics (*p* < 0.05). Gonadotroph counts were 8.3 ± 0.4, 9.4 ± 0.5, and 8.1 ± 0.4 cells in the 2–4, 5–10, and ≥11-year age groups, respectively, with males showing a higher overall count than females (9.1 ± 0.4 vs. 8.2 ± 0.3; *p* < 0.05). Overall cell size, cell area, cell volume, nucleus area, and nucleus volume were also greater in males than females (*p* < 0.05). The largest nucleus size, nucleus area, and nucleus volume were observed in camels ≥11 years of age, with values of 4.7 ± 0.1 µm, 18.5 ± 0.8 µm^2^, and 60.3 ± 4.1 µm^3^, respectively. Ultrastructural analysis further showed a significant sex difference in granule size (58.0 ± 0.7 vs. 52.2 ± 0.8 nm) and granule percentage (56.2 ± 3.6 vs. 43.5 ± 3.3% in males and females, respectively; *p* < 0.05). Serum FSH concentrations were higher in males than females (4.8 ± 0.1 vs. 4.2 ± 0.1 µIU/mL; *p* < 0.05), whereas LH concentrations were 1.2 ± 0.1 and 1.2 ± 0.1 µIU/mL in males and females, respectively. LH concentration was highest in 5–10-year-old males (1.3 ± 0.2 µIU/mL), while FSH concentration was highest in ≥11-year-old males (4.8 ± 0.2 µIU/mL). Pearson correlation analysis demonstrated significant positive associations between gonadotroph morphometric parameters and circulating gonadotropins, with correlations ranging from 0.52 to 0.74 for cell/nuclear measurements with LH and FSH. Overall, the findings demonstrate marked age- and sex-associated differences in gonadotroph morphology, ultrastructure, and circulating gonadotropin concentrations in dromedary camels, with males generally exhibiting greater gonadotroph dimensions and secretory granule characteristics. These structural and endocrine differences provide evidence of age- and sex-related variation in pituitary gonadotrophic activity, although the findings should be interpreted in light of the cross-sectional design and the undetermined reproductive status of female camels. Overall, these findings demonstrate pronounced age- and sex-associated differences in pituitary gonadotroph morphology, ultrastructure, and endocrine activity, with males generally exhibiting greater cellular and secretory characteristics. The findings provide novel morphological and endocrine evidence of reproductive aging and sexual dimorphism in the dromedary camel pituitary and may contribute to improved understanding of reproductive function across different life stages. However, interpretation should consider the cross-sectional design and the inability to determine the precise reproductive stage of female camels before slaughter.

## Simple Summary

This study examined whether reproductive aging and sex are associated with structural changes in pituitary gonadotrophs and circulating reproductive hormones in dromedary camels. A total of 90 clinically healthy camels were categorized into three age groups (2–4, 5–10, and ≥11 years), with equal numbers of males and females. Gonadotroph number and cellular dimensions differed among age groups and between sexes, with the 5–10-year-old camels showing the greatest gonadotroph cell counts. Males generally exhibited greater gonadotroph numbers and larger cellular dimensions than females. Cell and nuclear dimensions also varied with advancing age, while serum LH and FSH concentrations showed age- and sex-related differences. The observed associations between gonadotroph morphology and circulating LH and FSH suggest coordinated structural and endocrine changes across reproductive life stages. Overall, these findings provide new morphological and endocrine information on age- and sex-related variation in the camel pituitary gonadotrophic system, contributing to a better understanding of reproductive physiology in dromedary camels. Future studies incorporating longitudinal assessments and detailed reproductive and endocrine profiling may further clarify the mechanisms underlying age- and sex-related changes in camel pituitary function and their implications for reproductive performance.

## 1. Introduction

The dromedary camel (*Camelus dromedarius*) is an economically and culturally important livestock species in arid and semi-arid regions, providing milk, meat, transport, and draft power [1]. Despite its remarkable adaptation to harsh environmental conditions, its reproductive physiology remains less extensively studied than that of other domestic species [2]. Reproductive efficiency is a key determinant of productivity in camel husbandry and depends largely on coordinated regulation of the hypothalamic–pituitary–gonadal (HPG) axis [3,4]. Within this endocrine network, the anterior pituitary gland plays a central role through the secretion of gonadotropins, luteinizing hormone (LH) and follicle-stimulating hormone (FSH), which regulate gonadal development, steroidogenesis, follicular growth, ovulation, and spermatogenesis [5].

Gonadotropins are synthesized and secreted by specialized endocrine cells known as gonadotrophs, located in the pars distalis of the adenohypophysis [6]. The morphology, abundance, and secretory activity of gonadotrophs are closely associated with the physiological and reproductive status of animals [7]. Changes in gonadotroph structure may accompany alterations in hormone synthesis and secretion and can therefore provide useful morphological information about pituitary endocrine function. In several mammalian species, age-related changes in pituitary gonadotroph populations have been reported [8,9,10], including alterations in gonadotroph number, cell size, and cellular activity associated with reproductive maturation, peak reproductive activity, and reproductive senescence [10,11]. Sexual dimorphism in pituitary endocrine cells has also been documented, with differences in gonadotroph number, cellular dimensions, nuclear morphology, and secretory granule characteristics reported between males and females in several species [12,13,14,15]. Such age- and sex-related changes may influence circulating gonadotropin concentrations and reproductive performance [16,17,18].

The activity of the HPG axis is also influenced by physiological reproductive status, including the estrous cycle, pregnancy, and lactation, which can alter gonadotroph morphology and circulating LH and FSH concentrations through changes in hypothalamic and gonadal feedback. In addition, dromedary camels are seasonal breeders, and reproductive activity and circulating reproductive hormones vary according to season and environmental conditions. Seasonal changes in photoperiod, temperature, nutrition, and associated hypothalamic–pituitary–gonadal activity may therefore contribute to variation in gonadotroph function and circulating gonadotropin concentrations. These factors are important when interpreting age- and sex-related differences in camel reproductive endocrinology and should be considered when evaluating pituitary gonadotroph characteristics.

Recent research has provided evidence of considerable plasticity in camel pituitary endocrine cells. Our previous studies demonstrated age- and sex-related alterations in thyrotroph morphology and circulating thyroid hormone concentrations [3] and further identified differences in thyrotroph morphometry and thyroid hormone dynamics between lactating and non-lactating female camels [4]. Together with evidence from other mammalian species [8,9,10,11,12,13,14,15,16,17,18], these findings indicate that pituitary endocrine cells can undergo structural and functional adaptations in response to changing physiological demands. However, specific information on the gonadotrophic component of the pituitary in dromedary camels remains limited. In particular, the combined effects of age and sex on gonadotroph number, morphology, and ultrastructure, and their relationships with circulating LH and FSH concentrations, have not been comprehensively characterized. Consequently, the structural and endocrine characteristics of the HPG axis across different reproductive life stages remain poorly understood in this species.

Characterizing gonadotroph morphology and its relationship with circulating gonadotropins may have practical implications beyond understanding pituitary biology. Establishing age- and sex-related morphological and endocrine patterns could provide baseline indicators for assessing reproductive maturity, identifying age-associated reproductive changes, and improving interpretation of reproductive endocrine disorders in breeding animals. Such information may ultimately support more informed selection and management of breeding camels, reproductive monitoring, and veterinary approaches to disorders involving gonadal or pituitary dysfunction. Although gonadotroph morphology alone cannot be considered a direct measure of fertility, its association with circulating LH and FSH may provide a useful complementary indicator of reproductive endocrine status when interpreted together with reproductive and clinical parameters.

Therefore, the present study aimed to determine the effects of age and sex on pituitary gonadotroph number, morphology, and ultrastructural characteristics and to evaluate their relationships with circulating LH and FSH concentrations in dromedary camels. We hypothesized that gonadotroph morphology and circulating LH and FSH concentrations would differ according to age and sex, and that gonadotroph morphometric characteristics would be positively associated with circulating gonadotropin concentrations, reflecting coordinated structural and endocrine variation within the HPG axis.

## 2. Materials and Methods

### 2.1. Experimental Animals

The present study was conducted on 90 clinically healthy dromedary camels (*Camelus dromedarius*) of the Brela breed from March 2023 to February 2024. Animals were sourced from a single commercial camel farm supplying municipal abattoirs, ensuring uniform management, feeding practices, and environmental conditions. All camels were maintained under an extensive grazing system typical of the Cholistan Desert, with free access to natural pasture and seasonal browse species, supplemented with concentrate feed, mineral mixtures, and household crop residues when available. Animals were herded during the day and housed in traditional open-air enclosures at night. The sample size was determined by the availability of eligible animals meeting the predefined inclusion criteria during the study period, while maintaining a balanced factorial design across age and sex groups. A total of 90 camels, comprising 15 males and 15 females within each of the three age groups, were included to ensure equal representation and facilitate comparisons of age- and sex-related effects on the measured morphometric and endocrine variables. An a priori statistical power analysis was not performed because animal availability was constrained by the commercial abattoir-based sampling design. Therefore, the sample size should be considered a limitation of the study, and the statistical findings should be interpreted with consideration of the possibility of limited power to detect small or subtle effects.

The health status of the animals was assessed through clinical examination, including evaluation of body condition, rectal temperature, respiratory rate, heart rate, appetite, and general behavior. Anamnestic information obtained from farm records and owners was used to exclude animals with a recent history of systemic disease, reproductive disorders, or treatment with hormonal or metabolic drugs. Only animals exhibiting normal clinical and reproductive characteristics and free from gross pathological lesions were included in the study. Specifically, the inclusion criteria comprised clinically healthy camels within the predefined age categories, with normal body condition and general behavior, no recent history of systemic or reproductive disease, no recent hormonal or metabolic treatment, and normal gross reproductive anatomy at postmortem examination. Animals showing clinical signs of disease, a history of reproductive abnormalities or hormonal/metabolic treatment, or gross pathological lesions affecting the reproductive tract were excluded. Although clinical examinations were performed, no biochemical assessments of health biomarkers (e.g., hematological profiles or liver and kidney function tests) were conducted, which is acknowledged as a limitation of the study. Pre-slaughter ultrasonographic examination and endocrine profiling were not performed because the animals were obtained through a commercial abattoir and were not available for longitudinal reproductive monitoring before slaughter. Consequently, baseline reproductive endocrine status and specific physiological stages such as follicular, luteal, pregnant, or anestrous status could not be established before slaughter. Although gross postmortem examination of the reproductive tract was performed, these observations were used only to exclude animals with obvious reproductive abnormalities and could not accurately define the physiological reproductive stage. Therefore, reproductive stage may have contributed to variation in pituitary morphology and circulating LH and FSH concentrations, and this potential source of variation should be considered when interpreting the findings.

Animals were categorized into three age groups representing distinct reproductive life stages: 2–4 years (peri-pubertal to early reproductive stage), 5–10 years (prime reproductive maturity), and ≥11 years (late maturity or senescence). Each age group consisted of 15 males and 15 females, resulting in a total of 30 animals per group. Age was determined through dental examination and verified using farm management records and owner-provided information. These age categories were selected to represent key reproductive stages—sexual maturation, peak reproductive activity, and late reproductive life—and to enable evaluation of age-related changes in pituitary gonadotroph morphology and circulating LH and FSH concentrations. This classification was based on established reproductive milestones in dromedary camels, where females typically attain puberty between 3 and 4 years of age.

### 2.2. Assessment of Reproductive Status

To reduce potential confounding associated with reproductive status, all animals underwent reproductive tract examination immediately after slaughter. In females, the ovaries and uterus were examined for the presence of follicles, corpora lutea, and other indicators of ovarian cyclicity, whereas in males, testicular size and consistency, together with characteristic signs of rutting behavior (including poll gland enlargement and neck muscle hypertrophy), were assessed under the supervision of an experienced veterinarian specializing in large-animal reproduction. Only animals exhibiting normal reproductive anatomy and free from gross pathological lesions were included in the study. This combined approach of age verification, clinical evaluation, and postmortem reproductive assessment improved the biological characterization of the study population and reduced the likelihood of including animals with overt reproductive abnormalities. However, because animals were obtained from a commercial abattoir, the precise reproductive status of female camels (e.g., follicular or luteal phase, pregnancy status, or anestrus) could not be determined using ultrasonography or endocrine profiling before slaughter. Consequently, although gross postmortem examination confirmed normal reproductive anatomy, it could not accurately define the physiological reproductive stage. Therefore, variation associated with reproductive status may have contributed to differences in pituitary morphology and circulating LH and FSH concentrations and should be considered when interpreting the findings.

### 2.3. Tissue Processing and Immunohistochemical Analysis

Immediately after slaughter, the pituitary glands were carefully excised and immersed in Bouin’s fixative within approximately 15–20 min of death to minimize postmortem autolytic changes. Serial midsagittal sections (4 μm) were cut using a rotary microtome (Leica RM-2235, Leica Biosystems, Nussloch, Germany) and mounted on Poly-L-Lysine-coated glass slides (Sigma, St. Louis, MO, USA). The prepared slides were maintained in dust-free storage boxes for one week before immunohistochemical evaluation. Prior to staining, sections were treated with Lugol’s iodine solution for 5 min and subsequently immersed in 2.5% sodium thiosulphate for 1 min to eliminate residual iodine staining. Endogenous peroxidase activity was suppressed by incubation in 3% hydrogen peroxide for 15 min. Following deparaffinization in xylene, sections were further exposed to 0.3% hydrogen peroxide prepared in methanol for 30 min. To minimize non-specific antibody binding, the slides were incubated with normal goat serum for 30 min at room temperature in a humidified chamber. Polyclonal antibodies (pAbs) were initially evaluated during the immunohistochemical (IHC) screening procedure using biotinylated goat anti-rabbit IgG. Their final characterization was carried out on camel pituitary glands fixed in Bouin–Hollande solution. Following routine histological processing, 4-µm sections were prepared and mounted on poly-L-lysine-coated slides. The sections were subsequently deparaffinized, rehydrated through a graded ethanol series, and rinsed in phosphate-buffered saline (PBS; pH 7.2) for 10 min. To inhibit endogenous peroxidase activity, sections were treated with 0.3% hydrogen peroxide in methanol for 30 min. Non-specific binding sites were blocked by incubation with normal goat serum for 30 min at room temperature. The sections were then incubated in a humidified chamber at 37 °C for 120 min with 100 µL of rabbit anti-camel LH antibody (1:50 dilution), kindly provided by Dr. Yves Combarnous (CNRS–INRA, Nouzilly, France). LH immunohistochemistry was selected because a well-characterized rabbit anti-camel LH antibody was available and had previously been validated for the identification and morphometric evaluation of gonadotroph cells in camel pituitary tissue. A camel-specific antibody suitable for FSH immunohistochemistry was not available; therefore, FSH was assessed only by plasma ELISA, whereas LH immunostaining was used for the histomorphometric characterization of gonadotroph cells. After thorough washing in PBS, the sections were incubated with biotinylated goat anti-rabbit IgG for 30 min, followed by incubation with a streptavidin–peroxidase complex for an additional 30 min. Between each step, slides were washed three times in PBS for 5 min each. Immunoreactivity was visualized using diaminobenzidine (DAB) as the chromogenic substrate. Unless otherwise specified, all procedures were performed at room temperature. Negative control sections were processed in parallel by substituting the primary antibody with normal rabbit serum. Luteinizing hormone (LH)-immunoreactive cells were identified and evaluated based on the intensity and distribution of the staining reaction.

### 2.4. Electron Microscopy

Pituitary samples were cut into small pieces (a few millimeters in size) and fixed by immersion in 5% glutaraldehyde prepared in phosphate buffer (pH 6.8). Samples were post-fixed in 1% osmium tetroxide for 18 h at room temperature, rinsed thoroughly in running tap water, dehydrated through a graded ethanol series, and further treated with absolute acetone. The tissue was then embedded in Spurr’s resin. Polymerized resin blocks were trimmed and faced using a scalpel blade and glass knife before preparing ultrathin sections (120 nm) with an RMC MT-7000 ultramicrotome (Research and Manufacturing Company, Tucson, AZ, USA)). Sections were mounted on nickel grids (GC 200 mesh/inch), stained with 5% uranyl acetate and lead citrate in sodium hydroxide, and examined using a JEOL JEM-1010 transmission electron microscope (Japan Electron Optics Laboratory, Tokyo, Japan) at the National Institute of Biotechnology and Genetic Engineering (NIBGE), Faisalabad, Pakistan. Micrographs were captured from representative, well-preserved regions of the anterior pituitary showing minimal processing artifacts. Gonadotroph cells were identified based on established ultrastructural characteristics and were selected only when cellular and nuclear morphology was well preserved. Morphometric measurements were performed on representative cells visible within five electron micrographs obtained from each gland. All measurements were performed by a single experienced observer using a calibrated image analysis system based on the microscope scale bar to ensure measurement consistency. Micrographs were captured at a final magnification of 24,000×. For each pituitary gland, five non-overlapping representative fields containing well-preserved gonadotrophs were randomly selected from different regions of the pars distalis to minimize sampling bias, and biometric measurements were performed on these images using the predefined morphometric criteria. This sampling strategy was adopted consistently for all animals to ensure standardized comparisons among experimental groups.

### 2.5. Quantitative Morphometric Assessment

Morphometric evaluation was performed using a compound microscope, with measurements restricted to gonadotrophs exhibiting complete cellular profiles and clearly defined, non-immunoreactive nuclei. Pituitary sections from all 90 animals (15 males and 15 females within each of the three age groups) were subjected to morphometric analysis. For each sample, five histological sections were examined, and ten randomly selected microscopic fields were evaluated per section. The morphometric parameters assessed included gonadotroph number, cell diameter, cell area, cell volume, nuclear diameter, nuclear area, and nuclear volume. Cell and nuclear diameters (µm) were measured at two perpendicular axes—the maximum diameter and a second diameter taken at a 90° angle—using ImageJ software (ImageJ 1.44P, National Institutes of Health, Bethesda, MD, USA) for image calibration and linear measurements. AutoCAD 2004 was subsequently used to verify and accurately delineate cell and nuclear boundaries and to facilitate geometric measurements following the standardized morphometric protocol described by Jaspal et al. [3]. Cell and nuclear areas were subsequently calculated using the formula (*A* = *πr*^2^), while volumes were estimated using (*V* = 4/3 *πr*^3^), following the methodology described by Jaspal et al. [3].

### 2.6. Serum Luteinizing Hormone (LH) and Follicle-Stimulating Hormone (FSH) Assays

Blood samples were collected by direct jugular venipuncture to minimize potential variations in circulating hormone concentrations associated with different sampling sites. Approximately 10 mL of blood was drawn into heparinized tubes and centrifuged at 2500 rpm for 10 min. The resulting plasma was carefully separated and stored at −20 °C until hormonal analysis. Plasma concentrations of luteinizing hormone (LH; IU/mL) and follicle-stimulating hormone (FSH; IU/mL) were quantified using commercially available ACTIVE^®^ ELISA kits (DSL-10-4600 for LH and DSL-10-4700 for FSH; Diagnostic Systems Laboratories, Webster, TX, USA), according to the manufacturer’s instructions. Although these commercial ELISA kits were not specifically validated for dromedary camel plasma, they have been widely used for gonadotropin determination in veterinary species because of the high structural conservation of LH and FSH across mammals. Furthermore, the low intra- and inter-assay coefficients of variation obtained in the present study indicate acceptable analytical precision. Nevertheless, the lack of species-specific validation should be considered a limitation when interpreting the absolute hormone concentrations. The analytical sensitivity of both assays was 0.10 mIU/mL. The intra-assay coefficient of variation ranged from 5.6% to 6.8% for LH and from 1.8% to 3.4% for FSH, whereas the inter-assay coefficient of variation ranged from 5.3% to 7.8% for LH and from 2.0% to 5.4% for FSH, indicating acceptable assay precision and reproducibility. Because LH and FSH secretion is pulsatile and may be influenced by circadian and physiological rhythms, a single blood sample provides only a snapshot of circulating hormone concentrations rather than a complete endocrine profile. Blood collection was performed under standardized sampling conditions; however, serial blood sampling to characterize pulsatile hormone secretion was not feasible because the animals were obtained from a commercial abattoir.

### 2.7. Statistical Analysis

Data were expressed as mean ± standard error of the mean (SEM). Prior to statistical analysis, data were tested for normality using the Shapiro–Wilk test and for homogeneity of variances using Levene’s test. Morphometric and hormonal variables were analyzed using a two-way factorial analysis of variance (ANOVA) to evaluate the main effects of age group (2–4, 5–10, and ≥11 years), sex (male and female), and their interaction (Age × Sex). When significant main or interaction effects were detected, mean comparisons were performed using Tukey’s honestly significant difference (HSD) post hoc test. In addition, Pearson correlation analysis was performed to assess the relationships between gonadotroph morphometric parameters and serum LH and FSH concentrations. Statistical significance was accepted at *p* < 0.05. Exact *p*-values, F-statistics, Pearson correlation coefficients (r), and effect sizes (partial eta squared, η^2^p), where applicable, are reported in the Results. All statistical analyses were performed using IBM SPSS Statistics version 21.0 (IBM Corp., Armonk, NY, USA).

## 3. Results

**Figure 1:**
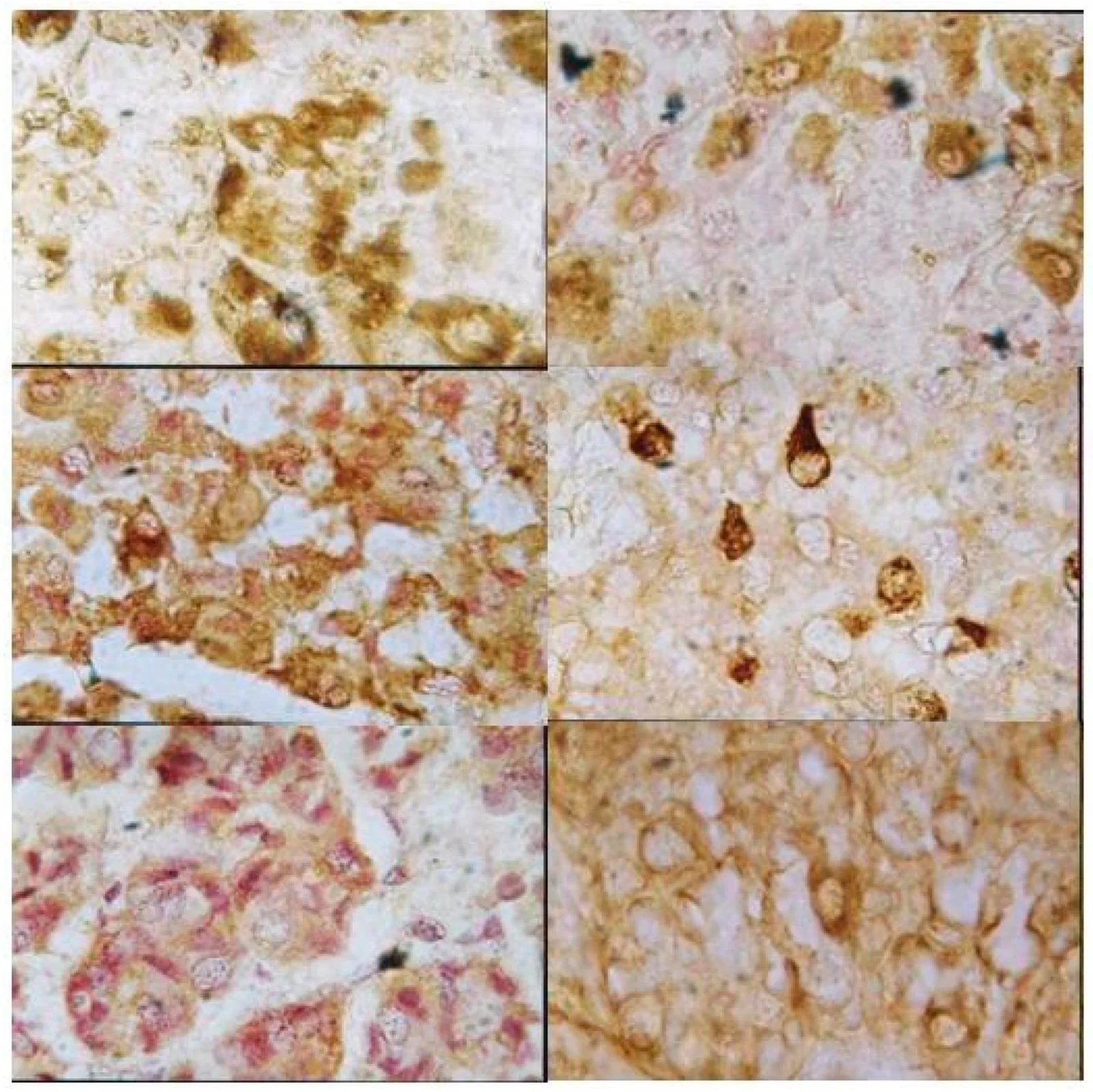
Representative photomicrographs of luteinizing hormone (LH)-immunoreactive gonadotrophs in the pars distalis of the adenohypophysis of dromedary camels (*Camelus dromedarius*) from different age groups and sexes. Immunohistochemical staining was performed using the streptavidin–peroxidase method (original magnification ×500). Male, 2–4 years: numerous polygonal LH-immunoreactive gonadotrophs arranged in clusters, with some cells exhibiting relatively large nuclei. Female, 2–4 years: clustered polygonal gonadotrophs, predominantly with small, regular nuclei. Male, 5–10 years: abundant polygonal to irregular gonadotrophs with small, irregular nuclei. Female, 5–10 years: fewer LH-immunoreactive gonadotrophs with narrow cytoplasm, irregular nuclei, and reduced cytoplasmic staining intensity. Male, ≥11 years: polygonal gonadotrophs with relatively regular nuclei. Female, ≥11 years: scattered polygonal gonadotrophs with irregular nuclei and lower apparent cellular density than younger females.

The morphometric characteristics of immunocytochemically stained gonadotrophs and circulating LH and FSH concentrations exhibited age- and sex-related variations (Table 1; Figure 1). Gonadotroph cell count was highest in camels aged 5–10 years, whereas cell size, cell area, cell volume, nuclear area, and nuclear volume generally increased with advancing age, reaching their highest values in the ≥11-year group. Overall, males exhibited significantly greater gonadotroph cell count, cell size, cell area, cell volume, and nuclear volume than females (*p* < 0.05). In contrast, serum FSH concentrations remained relatively stable across age groups and between sexes, whereas serum LH concentrations varied significantly with age and were highest in camels aged 5–10 years.

**Figure 2:**
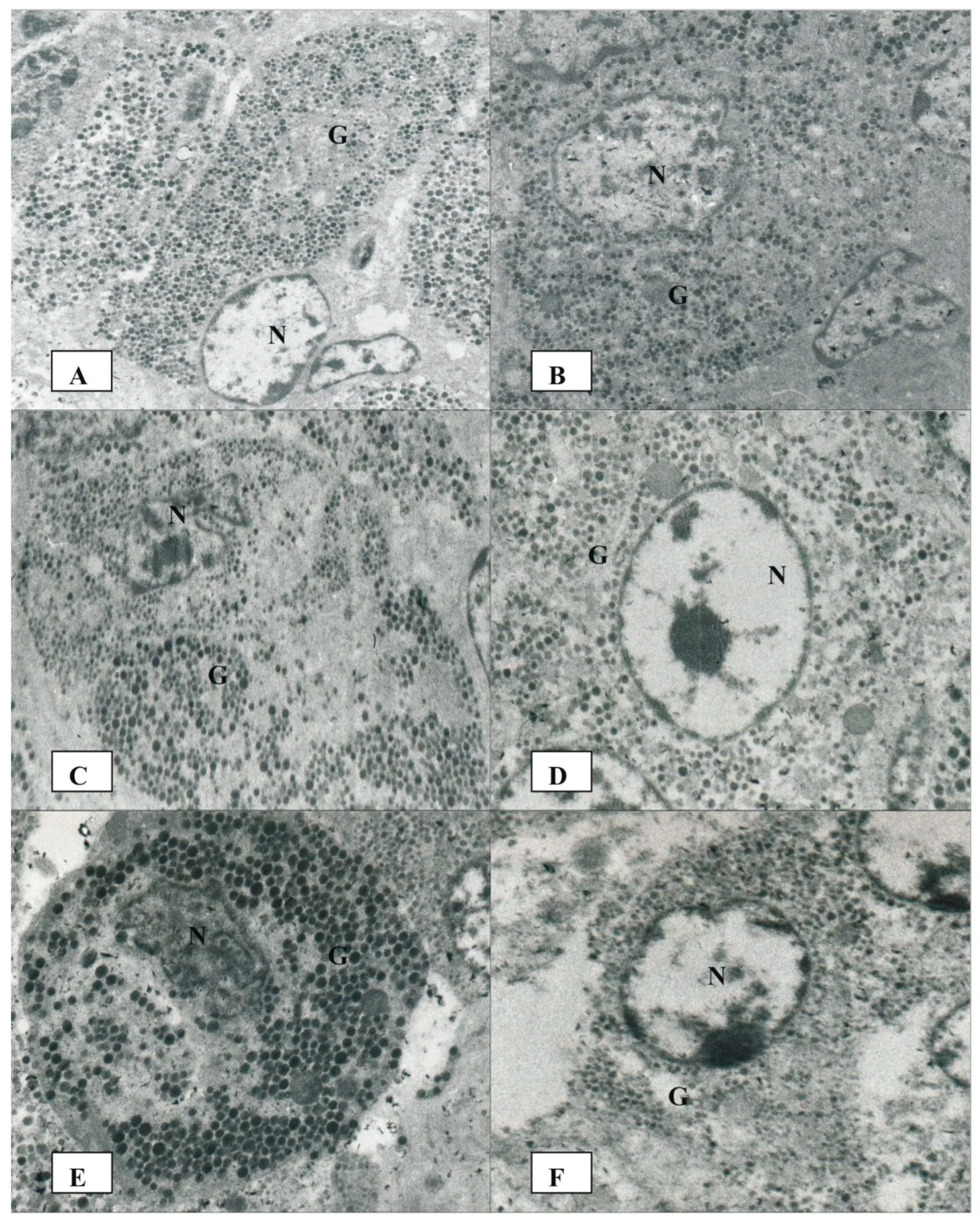
Representative transmission electron micrographs of gonadotroph cells in the pars distalis of the adenohypophysis of dromedary camels (*Camelus dromedarius*) from different age groups and sexes (original magnification ×2400). (**A**) Male, 2–4 years: gonadotroph with a moderately irregular nucleus and numerous secretory granules dispersed throughout the cytoplasm. (**B**) Female, 2–4 years: gonadotroph with a relatively small, irregular nucleus and abundant small secretory granules distributed within the cytoplasm. (**C**) Male, 5–10 years: gonadotroph with a centrally located nucleus and secretory granules of variable size arranged in distinct cytoplasmic regions. (**D**) Female, 5–10 years: gonadotroph with a large oval nucleus and relatively few cytoplasmic secretory granules. (**E**) Male, ≥11 years: gonadotroph with an irregular nucleus and abundant large secretory granules distributed throughout the cytoplasm. (**F**) Female, ≥11 years: gonadotroph with a relatively large irregular nucleus and secretory granules of variable size, with small granules predominating over larger granules. G, secretory granules; N, nucleus.

Ultrastructural morphometric analysis also revealed age- and sex-dependent differences in gonadotroph morphology (Table 2; Figure 2). Cell size, granule size, cell area, cell volume, and granule percentage varied significantly among age groups (*p* < 0.05), whereas nuclear size, nuclear area, nuclear volume, cytoplasmic percentage, and nuclear percentage did not differ significantly. Granule size decreased in older camels, while males exhibited significantly greater overall granule size and granule percentage than females (*p* < 0.05). The remaining ultrastructural variables showed no consistent sex-related differences.

Pearson correlation analysis revealed significant positive associations among gonadotroph morphometric parameters and circulating gonadotropin concentrations (Table 3). Strong positive correlations were observed among the gonadotroph morphometric parameters, including cell size, cell area, cell volume, nucleus size, nucleus area, and nucleus volume (r = 0.70–0.96; *p* < 0.001). Serum FSH showed moderate to strong positive correlations with the morphometric variables (r = 0.59–0.74; *p* < 0.001), whereas LH exhibited moderate positive correlations with the morphometric parameters (r = 0.52–0.68; *p* < 0.001). A weak positive correlation was observed between serum FSH and LH concentrations (r = 0.38; *p* < 0.001).

## 4. Discussion

The present study demonstrates clear age- and sex-dependent alterations in pituitary gonadotroph morphology and serum LH and FSH concentrations in C. dromedarius, suggesting a dynamic structural–functional relationship within the hypothalamic–pituitary–gonadal (HPG) axis. These findings align with the broader concept that pituitary cell populations exhibit marked plasticity in response to physiological demands, as similarly reported in the hypothalamic–pituitary–thyroid (HPT) axis of camels [3]. Pearson correlation analysis further demonstrated significant moderate to strong positive correlations between FSH concentrations and gonadotroph morphometric parameters (r = 0.59–0.74; *p* < 0.001), particularly cell area and cell volume, suggesting that greater gonadotroph dimensions were associated with higher circulating FSH concentrations. However, these correlations indicate statistical associations rather than causal relationships, and the present study did not evaluate gene expression, hormone synthesis markers (e.g., LHβ and FSHβ), receptor abundance (e.g., GnRH receptor), cellular proliferation markers such as Ki-67, or intracellular signaling pathways.

Because studies specifically addressing gonadotroph morphology and its relationship with circulating gonadotropins in dromedary camels are limited, comparisons with other pituitary endocrine cell populations in this species provide useful contextual information. Our previous studies demonstrated age-, sex-, and physiological-state-related alterations in thyrotroph morphology and thyroid hormone profiles in dromedary camels [3,4]. These findings support the broader concept that anterior pituitary cell populations exhibit structural plasticity in response to changing physiological and endocrine demands. The present study extends this concept to gonadotrophs within the HPG axis, demonstrating significant age- and sex-related variation in gonadotroph morphology together with positive associations between gonadotroph dimensions and circulating LH and FSH concentrations. Although the HPT and HPG axes have distinct physiological functions and regulatory mechanisms, the findings from both systems suggest that pituitary endocrine cells may undergo structural remodeling in association with changing endocrine requirements. Importantly, the present findings should be interpreted independently of the thyrotroph studies because the mechanisms governing gonadotroph regulation were not directly investigated here.

The variation in gonadotroph cell count across age groups, with a peak in the 5–10-years group, is consistent with maximal reproductive endocrine activity during the prime reproductive phase. This pattern is biologically consistent with the expectation that gonadotroph populations may be more prominent during periods of active reproductive function and greater gonadotropic demand [10]. The subsequent decline in older camels may suggest age-related pituitary senescence or reduced hypothalamic stimulation, which has also been observed in other endocrine systems. This pattern closely parallels observations in thyrotroph populations, where younger camels (5–10 years) exhibited higher thyroidal activity and greater hormonal output, while older animals showed compensatory cellular enlargement despite reduced functional efficiency [3,4]. Such parallel adaptations may suggest a conserved endocrine aging mechanism across both HPG and HPT axes in camels.

Cellular hypertrophy observed in gonadotrophs, including increased cell size, area, and volume with age, may indicate enhanced secretory demand during reproductive maturity. Enlargement of pituitary cells has been associated with increased organelle development required for hormone synthesis [19]. Nevertheless, because intracellular organelles and hormone synthesis were not directly quantified in the present study, this mechanism should be considered a plausible interpretation rather than direct evidence of increased secretory activity. Similarly, in the HPT axis, non-lactating and older camels exhibited enlarged thyrotroph cell and nuclear dimensions, which were interpreted as compensatory hypertrophy in response to reduced hormonal output [20,21]. This suggests that pituitary hypertrophy in both gonadotrophs and thyrotrophs may represent a general compensatory mechanism to maintain endocrine output under reduced physiological efficiency. Such parallel adaptations suggest a conserved endocrine aging mechanism across both HPG and HPT axes in camels, possibly characterized by early-life functional optimization followed by late-life structural compensation. The positive correlations observed between cell size, cell area, cell volume, and circulating gonadotropin concentrations further support the close association between gonadotroph structural characteristics and endocrine function, although these relationships should not be interpreted as evidence of causality.

The present findings also demonstrated significant nuclear enlargement with age, particularly in older camels, which may be associated with increased transcriptional activity [22]. However, transcriptional activity was not directly assessed in the present study; therefore, the observed nuclear enlargement should not be interpreted as definitive evidence of increased gene transcription. Comparable nuclear changes have been reported in the camel HPT axis, where alterations in thyrotroph nuclear morphology were associated with changes in thyroid endocrine status [3,23]. Together, these observations suggest that nuclear hypertrophy is a shared adaptive response across pituitary cell types, which may reflect increased transcriptional demand, although this was not directly assessed in the present study.

The endocrine findings revealed distinct patterns for FSH and LH. Serum FSH concentrations were relatively stable across age groups, although males showed a significantly higher overall concentration than females (4.8 ± 0.1 vs. 4.2 ± 0.1 µIU/mL). The stability of FSH may be attributed to its primary role in maintaining basal gonadal function and gametogenesis, processes that require sustained endocrine support throughout adult life ([24,25]). Similar observations have been reported in several mammalian species, where circulating FSH levels exhibit limited fluctuations compared with LH because of the stronger inhibitory regulation exerted by inhibin and other gonadal feedback mechanisms [26]. The absence of significant sex-related differences further indicates that FSH secretion in camels is maintained within a relatively narrow physiological range despite differences in reproductive function between males and females. Pearson correlation analysis revealed significant positive correlations between circulating LH concentrations and gonadotroph morphometric parameters (r = 0.52–0.68; *p* < 0.001), particularly with nucleus size and nucleus volume. However, these correlations indicate an association between gonadotroph morphology and circulating LH concentrations rather than a direct causal relationship.

In contrast, LH concentrations showed greater variation, with the highest value observed in 5–10-year-old males (1.3 ± 0.2 µIU/mL). This age group corresponds to peak reproductive maturity, when gonadal activity, steroidogenesis, and reproductive performance are maximal. Elevated LH concentrations during this period likely reflect increased hypothalamic gonadotropin-releasing hormone (GnRH) stimulation and enhanced pituitary responsiveness, resulting in greater LH synthesis and secretion [27]. The subsequent decline in older animals may be associated with age-related reductions in GnRH pulsatility, diminished pituitary sensitivity, or alterations in gonadal steroid feedback, phenomena commonly reported during reproductive aging in mammals [28,29]. However, because LH secretion is pulsatile and only a single blood sample was collected from each animal, the present measurements should be interpreted as single-time-point concentrations rather than direct measures of sustained LH secretion. Serial blood sampling and GnRH stimulation testing were not feasible because the animals were obtained from a commercial abattoir. Therefore, the observed LH concentrations should not be interpreted as direct measures of sustained pituitary secretory activity. The age-related pattern of LH secretion observed in the present study is consistent with the morphometric findings, where gonadotroph abundance and morphometric characteristics were most pronounced during the middle-age reproductive phase. This relationship was further supported by Pearson correlation analysis, which revealed significant moderate positive correlations between circulating LH concentrations and gonadotroph morphometric variables (r = 0.52–0.68; *p* < 0.001), indicating that greater gonadotroph dimensions were associated with higher circulating LH concentrations. It should be noted that serum LH concentrations were determined from a single blood sample collected from each animal. Because LH secretion is inherently pulsatile, a single measurement may not fully reflect the dynamic endocrine activity of the hypothalamic–pituitary–gonadal axis. Furthermore, GnRH stimulation testing and repeated blood sampling were not performed; therefore, the observed serum LH concentrations should be interpreted as representative of a single physiological time point rather than sustained pituitary secretory activity. Consequently, the association between pituitary gonadotroph morphology and circulating LH concentrations should be interpreted with caution, and terms such as “enhanced gonadotroph activity” refer to the overall structural and endocrine associations observed rather than direct functional evidence of increased hormone secretion.

The generally higher LH concentrations observed in females compared with males may reflect the greater physiological variability associated with ovarian activity. In females, LH secretion is strongly influenced by estrogen-mediated feedback mechanisms that regulate follicular development, ovulation, and corpus luteum function [30]. Periodic positive feedback effects of estrogens can enhance LH synthesis and release [31], and may lead to higher circulating concentrations than those typically observed in males. However, because the reproductive stage of the female camels (e.g., follicular phase, luteal phase, pregnancy, or anestrus) was not standardized or determined before slaughter, part of the observed variation in LH concentrations may have been attributable to these physiological differences rather than sex alone. Therefore, the sex-related differences in LH should be interpreted with appropriate caution. Conversely, testosterone in males predominantly exerts negative feedback on the hypothalamic–pituitary–gonadal axis [32], contributing to more stable LH secretion. These findings suggest that circulating LH concentrations may be a more sensitive indicator of reproductive status and pituitary functional activity than FSH in dromedary camels, reflecting the dynamic endocrine adjustments required to support age- and sex-specific reproductive functions. A limitation of the present study is that the precise reproductive status of female camels, including the follicular or luteal phase, pregnancy status, or anestrus, was not determined before slaughter. Although postmortem examination of the reproductive tract confirmed grossly normal reproductive anatomy and the presence of ovarian structures indicative of reproductive activity, these observations could not accurately define the physiological reproductive stage. Because circulating LH and FSH concentrations and pituitary gonadotroph morphology are influenced by ovarian cyclicity and reproductive status, part of the variability observed among female camels may reflect these physiological differences rather than age or sex alone. Future studies incorporating reproductive ultrasonography together with endocrine profiling of progesterone and estradiol are warranted to better characterize reproductive status and further validate the present findings.

Ultrastructural analysis further revealed significant variation in gonadotroph cell and granule size across age groups, suggesting possible alterations in secretory dynamics. Reduced granule size in older camels may reflect impaired hormone packaging or secretion efficiency. In parallel, thyrotroph studies demonstrated that lactating camels exhibited increased cell counts but reduced intracellular hormone retention, suggesting enhanced secretory turnover [32]. This parallel supports the concept that both gonadotrophs and thyrotrophs may regulate secretory granule dynamics in response to physiological demand rather than maintaining a fixed storage capacity. Consistent with this interpretation, Jaspal et al. [3,4] reported marked alterations in thyrotroph morphology and granule distribution associated with changes in endocrine activity, suggesting adaptive secretory plasticity of anterior pituitary cells. The observed positive correlations between ultrastructural morphometric characteristics and circulating gonadotropin concentrations further support the concept that morphological alterations in gonadotrophs are accompanied by corresponding endocrine changes. However, because only circulating hormone concentrations and morphometric parameters were assessed, these relationships should be regarded as associative rather than evidence of direct functional regulation.

The higher granule percentage observed in males may suggest greater endocrine and metabolic activity, likely influenced by androgens. This finding is consistent with Jaspal et al. [4], who reported larger thyrotroph morphometry and higher T3 concentrations in male camels, which has previously been interpreted as reflecting greater endocrine and metabolic activity. Similarly, Jaspal et al. [3] demonstrated a close association between thyrotroph morphology and thyroid hormone secretion, supporting the concept that structural adaptations of pituitary cells reflect functional endocrine demands. Together, these findings suggest that androgen-mediated endocrine activity may enhance both reproductive and metabolic functions in male camels.

Pearson correlation analysis demonstrated significant positive associations between gonadotroph morphometric parameters and circulating LH and FSH concentrations, providing statistical support for the observed parallel structural and endocrine changes. However, correlation does not establish causation, and the present study did not evaluate gene expression, hormone synthesis, receptor abundance, or intracellular signaling pathways. In addition, the endocrine evaluation was limited to circulating LH and FSH concentrations. Other components of the hypothalamic–pituitary–gonadal axis, including testosterone, estradiol, progesterone, gonadotropin-releasing hormone (GnRH), and inhibin, were not assessed. Therefore, the endocrine mechanisms underlying the observed morphometric changes could not be comprehensively characterized. Furthermore, reproductive performance outcomes, including fertility, semen quality, ovulation, conception rate, and pregnancy outcome, were not evaluated. Therefore, the observed differences in gonadotroph morphology and circulating LH and FSH concentrations cannot be directly linked to reproductive success. Therefore, the proposed mechanisms underlying gonadotroph adaptation should be regarded as biologically plausible hypotheses rather than definitive mechanistic conclusions. In addition, because the study employed a cross-sectional design in which different animals representing different age groups were examined, the observed age-related differences should be interpreted as comparisons among age groups rather than true longitudinal developmental changes within the same individuals. Furthermore, although the overall sample size (n = 90) was adequate for the planned factorial comparisons, each age × sex subgroup comprised 15 animals. Consequently, the study may have had limited statistical power to detect relatively small or subtle differences, particularly for variables showing greater biological variability. Because the present study was limited to morphometric observations and circulating hormone measurements, it did not investigate the molecular mechanisms (e.g., receptor expression, gene expression, or intracellular signaling) underlying these associations. Future studies integrating morphometric, molecular, and functional endocrine analyses are required to elucidate the cellular mechanisms governing pituitary adaptation in dromedary camels. Specifically, gene expression, receptor expression, and molecular markers of hormone synthesis or cellular proliferation, such as LHβ, FSHβ, GnRH receptor, and Ki-67, were not assessed, limiting the mechanistic interpretation of the observed structural and endocrine associations. Future studies integrating morphometric, molecular, and functional endocrine analyses are required to elucidate the cellular mechanisms governing pituitary adaptation in dromedary camels. Another limitation of the present study is that direct reproductive performance was not assessed. Parameters such as fertility, semen quality, ovulation, conception rate, and pregnancy outcome were not available; therefore, the observed differences in gonadotroph morphology and circulating LH and FSH concentrations cannot be directly linked to reproductive success. The present findings should consequently be interpreted as evidence of age- and sex-related variation in pituitary reproductive endocrine characteristics rather than direct measures of reproductive performance. Future studies combining pituitary morphometry and endocrine profiling with longitudinal assessment of reproductive performance are needed to determine whether these structural and hormonal differences translate into functional differences in fertility.

## 5. Conclusions

In conclusion, gonadotroph morphology and ultrastructural characteristics in dromedary camels are significantly influenced by both age and sex, with adult and male animals generally exhibiting greater cellular development and granule content. These findings indicate age- and sex-dependent structural adaptations of pituitary gonadotrophs that are associated with corresponding variations in circulating LH and FSH concentrations, providing further insight into reproductive endocrine regulation in dromedary camels.

## Figures and Tables

**Table 1:** Morphometric parameters of immunocytochemically stained LH gonadotroph and serum LH and FSH concentration of camels (*Camelus dromedarius*) in different age groups.

Groups	2–4 Years			5–10 Years			≥11 Years			Overall Mean	
	Male	Female	Mean	Male	Female	Mean	Male	Female	Mean	Male	Female
**Count** **(Number)**	9.7 ± 0.6 ^b^	7.0 ± 0.4 ^c^	8.3 ± 0.4	12.1 ± 0.6 ^a^	6.0 ± 0.4 ^cd^	9.4 ± 0.5	5.5 ± 0.2 ^d^	10.8 ± 0.4 ^ab^	8.1 ± 0.4	9.1 ± 0.4 *	8.2 ± 0.3
**Cell size** **(µm)**	10.0 ± 0.3 ^a^	6.9 ± 0.3 ^a^	8.5 ± 0.3 ^B^	9.4 ± 0.4 ^a^	7.4 ± 0.3 ^b^	8.4 ± 0.3 ^B^	10.1 ± 0.2 ^a^	10.1 ± 0.4 ^a^	10.1 ± 0.2 ^A^	9.8 ± 0.2 *	8.0 ± 0.2
**Nucleus size** **(µm)**	3.8 ± 0.1	3.1 ± 0.1	3.5 ± 0.1 ^B^	4.1 ± 0.3	3.5 ± 0.1	3.8 ± 0.1B	4.9 ± 0.1	4.4 ± 0.1	4.7 ± 0.1 ^A^	4.3 ± 0.1	3.7 ± 0.1
**Cell area** **(µm^2^)**	82.6 ± 6.0 ^a^	41.4 ± 4.1 ^b^	62.0 ± 0.5 ^B^	75.2 ± 7.4 ^a^	45.9 ± 3.7 ^b^	60.6 ± 0.5 ^B^	81.8 ± 3.9 ^a^	85.3 ± 6.7 ^a^	83.6 ± 3.9 ^A^	79.9 ± 3.4 *	57.5 ± 3.6
**Nucleus area** **(µm^2^)**	12.3 ± 0.9	8.5 ± 0.9	10.4 ± 0.7 ^B^	15.8 ± 2.5	10.5 ± 0.9	12.9 ± 1.3 ^B^	19.6 ± 0.9	16.4 ± 1.3	18.5 ± 0.8 ^A^	15.7 ± 0.9 *	11.8 ± 0.7
**Cell volume** **(µm^3^)**	597.3 ± 64.5 ^a^	223.6 ± 35.7 ^b^	410.5 ± 43.9 ^B^	542.7 ± 81.3 ^a^	251.0 ± 29.5 ^b^	396.9 ± 46.9 ^B^	570.9 ± 40.2 ^c^	632.5 ± 30.7 ^a^	601.5 ± 41.8 ^A^	570.3 ± 36.7 *	369.0 ± 34.8
**Nucleus volume** **(µm^3^)**	34.9 ± 4.1	21.4 ± 3.9	28.0 ± 2.9 ^B^	56.8 ± 14.6	28.0 ± 20.8	42.3 ± 7.7 ^B^	66.9 ± 4.6	53.7 ± 6.6	60.3 ± 4.1 ^A^	52.8 ± 5.4 *	34.3 ± 3.19
**FSH** **(µIU/mL)**	4.7 ± 0.1	4.5 ± 0.1	4.6 ± 0.1	4.8 ± 0.2	4.2 ± 0.2	4.5 ± 0.1	4.8 ± 0.2	3.9 ± 0.1	4.3 ± 0.2	4.8 ± 0.1 ^c^	4.2 ± 0.10
**LH** **(µIU/mL)**	0.7 ± 0.1 ^c^	1.4 ± 0.1 ^ab^	1.1 ± 0.1	1.3 ± 0.2 ^a^	1.2 ± 0.0 ^abc^	1.4 ± 0.1	1.1 ± 0.0 ^abc^	1.1 ± 0.2 ^bc^	1.1 ± 0.3	1.2 ± 0.1	1.2 ± 0.10

abcd: the same letters on the mean in a row do not differ significantly at *p* < 0.05; * significantly different overall mean between male and female at *p* < 0.05. Mean values bearing "A" or "B" in a row differ significantly (*p* < 0.05)

**Table 2:** Morphometric parameters of gonadotroph ultrastructure of camels (*Camelus dromedarius*) of different age groups.

Parameters	2–4 Years			5–10 Years			≥11 years			Overall Mean	
	Male	Female	Mean	Male	Female	Mean	Male	Female	Mean	Male	Female
Cell size (μm)	8.6 ± 0.01 ^ab^	10.7 ± 1.3 ^a^	9.7 ± 0.7	9.5 ± 0.1 ^ab^	8.3 ± 0.9 ^ab^	8.9 ± 0.5	10.6 ± 0.6 ^a^	7.5 ± 0.7 ^b^	9.1 ± 0.8	9.6 ± 0.4	8.9 ± 0.6
Nucleus size (μm)	2.7 ± 0.46	3.3 ± 0.4	3.0 ± 0.3	3.8 ± 0.3	3.9 ± 1.7	3.8 ± 0.8	3.5 ± 0.2	3.4 ± 0.3	3.5 ± 0.2	3.3 ± 0.2	3.5 ± 0.5
Granule size (nm)	54.7 ± 1.10 ^c^	58.2 ± 0.8 ^b^	0.7 ± 0.08	54.6 ± 1.0 ^c^	53.3 ± 0.8 ^b^	53.9 ± 1.0 ^c^	64.7 ± 0.80	45 ± 1.50 ^d^	54.8 ± 1.2	58 ± 0.7	52.2 ± 0.8
Cell area (μm^2^)	59.1 ± 0.01 ^ab^	93.8 ± 22.1 ^a^	76.7 ± 13.0	72.2 ± 12.3 ^ab^	55.6 ± 7.6 ^ab^	63.9 ± 7.4	89.3 ± 10.2 ^a^	46.06 ± 8.97 ^b^	67.6 ± 11.4	73.7 ± 6.7	65.1 ± 10.2
Nucleus area (μm^2^)	6.1 ± 1.6	9.0 ± 2.3	7.6 ± 1.5	11.7 ± 1.9	17.2 ± 12.0	14.5 ± 5.5	10.1 ± 1.2	9.60 ± 2.12	9.8 ± 1.1	9.3 ± 1.2	11.9 ± 3.8
Cell Volume (μm^3^)	352.34 ± 72.70 ^ab^	712.3 ± 243.5 ^a^	532.3 ± 139.2	471.6 ± 119.7 ^ab^	316.4 ± 62.7 ^ab^	394.0 ± 69.7	641.4 ± 106.0 ^ab^	241.7 ± 70.32 ^b^	441.6 ± 105.9	488.4 ± 65.8	423.5 ± 104.9
Nucleus volume (μm^3^)	12.3 ± 5.3	21.5 ± 8.1	16.9 ± 4.8	30.9 ± 7.5	73.4 ± 61.3	52.1 ± 29.2	24.4 ± 4.3	23.23 ± 7.41	23.8 ± 3.8	22.5 ± 4.2	39.4 ± 19.9
Cytoplasm percentage	89.4 ± 2.5	89.9 ± 4.4	80.6 ± 0.3	89.9 ± 1.2	82.0 ± 11.0	86 ± 5.2	92 ± 2.2	92 ± 2.25	81.7 ± 8.2	90.4 ± 1.2	84.5 ± 4.3
Nucleus percentage	10.5 ± 2.5	10.6 ± 4.4	0.48 ± 0.1	10.09 ± 1.2	17.9 ± 11.0	14 ± 5.2	8 ± 2.6	18.29 ± 8.20	13.1 ± 4.4	9.5 ± 1.1	15.4 ± 4.3
Granule percentage	54.2 ± 5.3	44.4 ± 4.5	54.3 ± 5.4	62.7 ± 5.4	46.83 ± 9.25	54.7 ± 5.9	51.78 ± 8.10	39.44 ± 3.65	45.61 ± 4.84	56.2 ± 3.6	43.5 ± 3.3

abcd: the same letters on the mean in a row do not differ significantly at *p* < 0.05.

**Table 3:** Pearson correlation coefficients among gonadotroph morphometric parameters and circulating gonadotropin (LH and FSH) concentrations in dromedary camels (*Camelus dromedarius*).

Variable	Cell Size	Nucleus Size	Cell Area	Nucleus Area	Cell Volume	Nucleus Volume	FSH	LH
Cell size	1.000	0.78 (<0.001)	0.92 (<0.001)	0.73 (<0.001)	0.95 (<0.001)	0.70 (<0.001)	0.66 (<0.001)	0.52 (<0.001)
Nucleus size		1.000	0.75 (<0.001)	0.91 (<0.001)	0.72 (<0.001)	0.95 (<0.001)	0.59 (<0.001)	0.63 (<0.001)
Cell area			1.000	0.76 (<0.001)	0.96 (<0.001)	0.74 (<0.001)	0.71 (<0.001)	0.56 (<0.001)
Nucleus area				1.000	0.75 (<0.001)	0.94 (<0.001)	0.61 (<0.001)	0.65 (<0.001)
Cell volume					1.000	0.76 (<0.001)	0.74 (<0.001)	0.58 (<0.001)
Nucleus volume						1.000	0.63 (<0.001)	0.68 (<0.001)
FSH							1.000	0.38 (<0.001)
LH								1.000

Note: Values are Pearson correlation coefficients (r), with corresponding *p* values in parentheses. Correlations were calculated using n = 90 animals. All reported correlations were statistically significant (*p* < 0.001).

## Data Availability

The original contributions presented in this study are included in the article. Further inquiries can be directed to the corresponding authors.
